# Applying a typology of health worker migration to non-EU migrant doctors in Ireland

**DOI:** 10.1186/s12960-015-0042-2

**Published:** 2015-06-26

**Authors:** Niamh Humphries, Sara McAleese, Ella Tyrrell, Steve Thomas, Charles Normand, Ruairí Brugha

**Affiliations:** Department of Epidemiology and Public Health Medicine, Royal College of Surgeons in Ireland, 123 St. Stephen’s Green, Dublin 2, Ireland; Centre for Health Policy and Management, Trinity College Dublin, Dublin, Ireland

**Keywords:** Doctor migration, Migration typology, Medical workforce planning, Health workforce planning, Health human resources for health

## Abstract

**Background:**

Research on health worker migration in the Irish context has categorized migrant health workers by country or region of training (for example, non-EU nurses or doctors) or recruitment mechanism (for example, actively recruited nurses). This paper applies a new typology of health worker migrants – *livelihood*, *career-oriented*, *backpacker*, *commuter*, *undocumented and returner migrants* (European Observatory on Health Systems and Policies and WHO, vol. 2:129-152, 2014) – to the experiences of non-EU migrant doctors in Ireland and tests its utility for understanding health worker migration internationally.

**Methods:**

The paper draws on quantitative survey (*N* = 366) and qualitative interview (*N* = 37) data collected from non-EU migrant doctors in Ireland between 2011 and 2013.

**Results:**

Categorizing non-EU migrant doctors in Ireland according to the typology (European Observatory on Health Systems and Policies and WHO, vol. 2:129-152, 2014) offers insight into their differing motivations, particularly on arrival. Findings suggest that the *career-oriented migrant* is the most common type of doctor among non-EU migrant doctor respondents, accounting for 60 % (*N* = 220) of quantitative and 54 % (*N* = 20) of qualitative respondents. The authors propose a modification to the typology via the addition of two additional categories – the *family migrant* and the *safety and security migrant*.

**Conclusions:**

Employing a typology of health worker migration can facilitate a more comprehensive understanding of the migrant medical workforce, a necessary prerequisite for the development of useful policy tools (European Observatory on Health Systems and Policies and WHO, vol. 2:129-152, 2014). The findings indicate that there is some fluidity between categories, as health worker motivations change over time. This indicates the potential for policy levers to influence migrant health worker decision-making, if they are sufficiently “tuned in” to migrant health worker motivation.

## Background

### Understanding the health workforce

Understanding health worker motivation and the factors influencing retention and migration decisions are critical to the successful delivery of health services. Once the right people have been recruited into the health workforce, it is critical “to keep them engaged and committed” [1]*.* Understanding health worker motivation should be an ongoing exercise, as motivations can change over time [2].

Previous migration research has illustrated that migration decision-making is far more complex than the “binary ‘return home or stay’ scenario often depicted in the literature” [3]. Health worker migration is influenced by many factors, including those endogenous to the health system (such as a desire for better working conditions) and those exogenous to the health system (such as a desire for improved entitlements to citizenship and family reunification) [4–6]. Previous research with migrant health workers revealed that when considering their onward migration from Ireland, migrant nurses prioritized exogenous factors [4], while migrant doctors prioritized endogenous factors [6]. This has been highlighted in the literature, “not all workers will have the same mix of motives and goals, and the relative importance of particular values and work goals will change over time and situations” [7]. Even the pace at which the decision to emigrate is made varies, with some health workers deciding to leave relatively quickly, while others wait in the hope that things will improve [8]. This poses a significant challenge to those responsible for human resources for health in the destination country in terms of managing the stock of health professionals.

### Migration typology

Research on health worker migration has categorized migrant health workers according to the following: by their region of origin – non-EU migrant doctors [6, 9], non-EU migrant nurses [10]; by the region in which they completed basic training – international medical graduates [11], foreign-trained nurses [12], foreign healthcare professionals [13]; by their migration status – migrant nurses [4, 10]; or by their method of recruitment – internationally recruited nurses [14]. The various methods of categorizing professional migrants are discussed in detail by Iredale [15]. While these labels enable the identification of migrant health workers within the destination country, they provide no insights into the motivations for migration.

In contrast, the typology of migrant health workers proposed by Glinos and Buchan facilitates an understanding of the “varied and nuanced nature of health professional mobility” [16]. Despite the speed at which the “magnitude, directions and impact” [17] of health worker migration flows are changing, certain patterns and trends have emerged from research on health worker motivations and intentions. These patterns have the potential to enable health human resource planners and managers to gain a greater understanding of the migrant health workers within their workforce. The typology of EU health worker mobility and migration developed by Glinos and Buchan (see Table 1) categorizes migrant health workers by the factors underpinning their migration decision. Migrant health workers are not simply “units” migrating in response to the workforce needs of health systems globally [4] nor are they motivated solely by personal financial gain. Although these factors influence the choices made by migrant health workers, individual health workers also have personal and professional motivations that contribute to their migration decisions. The Glinos and Buchan typology [16] connects research and policy by providing policy makers with an evidence-informed tool to assist with health human resource management. Its value stems from the fact that “identifying and understanding the various types of mobile health professionals is a prerequisite to conceiving useful policy tools” [16].

**Table 1:** Typology of mobile health workers [16]

Typology	Description
Livelihood migrant	Migrates for improved salary and better standard of living. S/he may migrate to escape unemployment or job insecurity, for example, the “crisis escapee” migrating from countries hardest hit by the financial crisis. The purpose of migration is to settle abroad, either temporarily or permanently.
Career-oriented migrant	Travels for career development, perhaps to acquire training or to accelerate career progression. S/he is likely to migrate for a specific period of time to acquire qualifications and/or experience that will enhance his/her career back home.
Backpacker migrant	Works to travel and sees mobility as an opportunity. S/he tends to remain in each destination country for a short period of time.
Commuter migrant	Travels across border to work, perhaps on a daily/weekly basis, or for longer periods of time if the distance between host and destination country is greater.
Undocumented migrant	Works in the informal sector, perhaps in care work, and is likely to be working below his/her skill level.
Returner migrant	Migrates in reverse, returning to the home country. S/he may return when the outlook improves in the home country or if the migration experience does not match their expectations.

### Irish context

Ireland has a particularly high reliance on migrant health workers – in 2013, migrant doctors accounted for 34.3 % of all doctors registered to practise in Ireland [18]. This reliance stems from an inability to retain locally trained doctors in the system. Many migrant doctors in Ireland occupy “service posts” which offer little or no prospect of career progression or postgraduate training [6, 9]. These posts are unattractive to locally trained doctors. Previous research by the authors revealed that non-EU doctors are also frustrated with these posts and with the limited career progression opportunities in the Irish health system [6, 9].

### Aim of the paper

The aim of this paper is threefold: (i) to apply the categories proposed by Glinos and Buchan [16] to the Irish context, (ii) to consider how the typology correlates with and helps to explain the experiences and motivation of migrant health workers within a destination country health system and (iii) to consider how this information might enhance and support health workforce planning nationally and internationally. As a high-income destination country with a heavy reliance on migrant doctors (accounting for one in three doctors), Ireland is a good location in which to test the applicability of the typology.

## Methods

The Doctor Migration Project focused on non-EU migrant doctors in Ireland and was conducted between 2011 and 2013. The project sought to identify respondents’ initial motivation for migration, their experiences of living and working in Ireland and their plans for the future. The project employed a sequential mixed methods approach, generating both qualitative and quantitative data from interviews and survey of non-EU migrant doctors in Ireland. A mixed methods approach was considered appropriate in order to enable the authors to better understand and evaluate the complexity of health professional migration [18].

Although there are limited data available on the non-EU medical workforce in Ireland, the authors sought to ensure that both interview and survey respondents were representative of the wider migrant medical workforce^1^. As shown in Table 2, those surveyed and interviewed shared key characteristics with international medical graduates (IMGs) registered in Ireland, particularly in terms of gender and current grade within the health system. In terms of country of training, some countries are over-represented and others under-represented, and these are noted in the limitations section of the paper.

**Table 2:** Profile of all International Medical Graduates (IMGs) registered to practise in Ireland, survey and interview respondents [32]

	International medical graduates (IMGs) registered in Ireland (2012) [32]	Survey respondents 2013	Interview respondents 2011
Gender	34.4 % female	30 % female (108)	35 % female (13)
Country of training	Pakistan 1200 (21 %)	Pakistan 59 (16 %)	Pakistan 10 (27 %)
	South Africa 768 (14 %)	South Africa 43 (12 %)	South Africa 1 (3 %)
	Sudan 527 (9 %)	Sudan 29 (8 %)	Sudan 8 (22 %)
	India 467 (8 %)	India 43 (12 %)	Nigeria 4 (11 %)
		Nigeria 40 (11 %)	India 3 (8 %)
Grade in Irish health system	General register 3482 (62 %)	213 junior hospital doctors (58 %)	24 junior hospital doctors (65 %)
	Specialist register 1468 (26 %)	103 consultants + GPs (28 %)	7 consultants + 2 GPs (24 %)
Totals	5715 IMGs	366 respondents	37 respondents

*GP* general practitioner

### Qualitative data

In-depth interviews were conducted with 37 non-EU migrant doctors working in Ireland in 2011; interview respondents were non-EU migrant doctors who had trained and/or were born in a non-EU country. At the time of interview, 33/37 respondents were working in the Irish health system, 2 had recently ceased working and 2 were seeking work in the Irish health system. Qualitative respondents were recruited in several ways: (i) via the Irish Medical Directory, (ii) via an advertisement in the Irish Medical Times, (iii) via an NGO working with immigrants in Ireland and (iv) participants in a previous study on non-EU doctors in Ireland. Interviews involved a discussion of respondents’ initial decision to migrate to Ireland, their employment situation pre-migration and the decision and mechanics of migration. Respondents were then asked about their experience in the Irish health system, specifically whether their pre-migration expectations had been met. Finally, respondents were asked to reflect on their future migration plans, whether they intended to remain in Ireland, return home or migrate onwards.

### Quantitative data

An online quantitative survey with non-EU migrant doctors was conducted in April/May of 2013. The survey contained 70 questions (not all of which were answered by all respondents) which were informed by the qualitative research findings [6, 9]. The survey included questions about the decision to migrate, immigration and registration processes and first employment post in Ireland. Respondents were asked about their current post and their experiences of training and working hours. The final section of the survey focused on respondent’s plans for the future – whether they intended to remain in Ireland, return home or migrate elsewhere. The survey also collected basic demographic information from respondents, as well as information about their current grade within the Irish health system and their year of arrival.

A total of 3009 non-EU migrant doctors in Ireland, registered in the Medical Council of Ireland’s Register of Medical Professionals, received an email inviting them to participate in the online survey (hosted by SurveyMonkey). This sampling frame represents 68 % of non-EU migrant doctors registered with the Medical Council of Ireland for whom a valid email address was available. The response rate was 16 % (483 non-EU migrant doctors). When partial responses were excluded, 366 non-EU migrant doctor survey respondents were included for analysis. Not all respondents answered all survey questions, so the overall number (*N*) will vary slightly from question to question.

### Data analysis

The qualitative and quantitative data were collected and analysed separately. This analysis represents an attempt to integrate or triangulate [19, 20] the data and to consider the extent to which the typology is relevant to experiences of respondent non-EU migrant doctors in Ireland. A similar approach was used previously by the authors in a study on migrant nurses in Ireland [10].

In the findings, respondents who participated in the in-depth interviews are referred to as “Doctor” and are numbered according to their study identification number, for example, “Doctor 35”. The open-ended responses from the survey are referenced as survey respondents and their study identification number and year of arrival are also included, for example, “Survey Respondent 326, 2010”.

#### Applying the typology

A typology was conceived at the beginning of the Doctor Migration Project that focused primarily on length of stay – the possibly permanent migrant doctor, probably temporary migrant doctor and probably temporary migrant medical student. This typology sought to categorize migrants as temporary or permanent, although implying a level of fluidity between categories. From a destination country perspective, the decision of migrant health workers to stay or leave the country is of primary concern. However, the process of data collection revealed a more complex migration process and necessitated a re-consideration of this typology. In the interim, Glinos and Buchan [16] developed a typology of health worker mobility. The authors have retrospectively analysed the qualitative and quantitative data from the Doctor Migration Project to assess the extent to which the Glinos and Buchan typology resonates with the experiences of respondent non-EU migrant doctors in Ireland. In so doing, the authors aim to validate the proposed typology and to contribute to its further development.

#### Connecting the data with the typology

Two authors (NH and SMcA) reviewed respondents’ answers to the question “tell me about your decision to come and work in Ireland” within the qualitative interview. The 37 respondents were then categorized according to the typology proposed by Glinos and Buchan [16]. Categorization considered only the major reason for migration. Where a respondent had migrated to Ireland on more than one occasion, they were categorized on the basis of their most recent arrival.

Survey respondents were categorized as *livelihood*, *career-oriented or backpacker migrants* on the basis of their responses to the question: “How important were each of the following factors in your decision to migrate to Ireland? [for family reasons, to obtain postgraduate medical qualifications, safety/security, wanted to live abroad, career progression, for higher salary, to obtain basic medical qualifications, other]”. Respondents were categorized as *commuter migrants* if they indicated that they worked in Ireland for less than 12 months per year. Respondents were categorized as *returner migrants* if they stated that their intention on arrival was to return home (see Table 3). The quantitative categories are not mutually exclusive, and there is overlap between some of the proposed categories (see Table 4). Once categorized according to the typology, the open-ended comments of respondents were reviewed and used to help to illustrate the categories. Particularly important in this regard were respondents’ answers to the final question in the online survey, “do you have any further comments about doctor migration to Ireland”.

**Table 3:** Connecting qualitative data with the Buchan and Glinos typology [16]

	Quantitative survey (*N* = 366)^a^	Qualitative interviews (*N* = 37)
Glinos and Buchan typology of migrants		
Career-oriented migrant	220 (60 %)	21 (57 %)
Livelihood migrant	49 (13 %)	3 (8 %)
Backpacker migrant	46 (13 %)	2 (5 %)
Commuter migrant	46 (13 %)	0
Undocumented migrant	0	0
Potential returner migrant	164 (45 %)	0
Family migrant^b^	62 (17 %)	9 (24 %)
Safety and security migrant^b^	73 (20 %)	2 (5 %)

^a^Respondents were assigned to more than one category

^b^New categories

**Table 4:** Connecting quantitative data with the typology [16]

Glinos and Buchan	Non-EU migrant doctor survey respondents
Livelihood migrant	Rated “for higher salary” as very important in the decision to migrate to Ireland.
Career-oriented migrant	Rated “to obtain postgraduate medical training” as very important in the initial decision to migrate to Ireland.
Backpacker migrant	Rated “wanted to live abroad” as very important in the decision to migrate to Ireland.
Commuter migrant	Indicated, in the “circular migration” section of the survey, that s/he works in Ireland for less than 12 months each year.
Undocumented migrant	No undocumented migrants were identified via the survey.
*Potential* returner migrant	Indicated that the intention on arrival was to return home.
Family migrant^a^	Rated “for family reasons” as very important in the initial decision to migrate to Ireland.
Safety and security migrant^a^	Rated “safety/security” as very important in the initial decision to migrate to Ireland.

^a^New categories

### Research findings

The qualitative and quantitative data show that the category most non-EU migrant doctor respondents align to is that of the *career-oriented migrant* (see Tables 3 and 4). The following section will discuss each category within the typology in turn, presenting quantitative and qualitative data from the Doctor Migration Project, before presenting the two new categories that have emerged from this analysis.

### Career-oriented migrant

*Career-oriented migrants* rated “to obtain postgraduate medical training” as very important in their initial decision to migrate to Ireland. According to these definitions, the *career-oriented migrant* was by far the most common type of migrant doctor – accounting for 60 % of survey respondents (220/366). The *career-oriented migrant* was also the most common category among interview respondents (21/37).

The *career-oriented migrant* had a clearly articulated goal on arrival in Ireland and that was to obtain postgraduate training and achieve career progression:

“I wasn’t focused on money… my main objective was training… I am here to get… my higher training and then I can make money afterwards when I go back.” (Doctor 31).

“I didn’t have any major expectations when I came in; I just wanted to do my exams, get my training and go home.” (Doctor 19).

The *career-oriented migrant* doctor had clear expectations of the Irish health system, that is, that s/he could come for a defined period of time, obtain postgraduate training and then move on, perhaps returning home. There is overlap between the two categories, with 89/220 *career-oriented migrants* also *potential returner migrants*. Survey respondents outlined how these expectations had been met, while highlighting the challenges involved in achieving them:

“Despite different obstacles, Ireland has provided me with opportunities to excel in my field.” (Survey Respondent 122, 1991).

“I achieved my goal partially but with great personal effort and more luck as the doors of training jobs remained closed.” (Survey respondent 202, 2001).

“Good experience and opportunity to train but… bone breaking work hours and unnecessary secretarial work can definitely takes a toll on even the most brilliant enthusiastic NCHD^2^.” (Survey Respondent 64, unknown year).

Even reflections by the *career-oriented migrants* on their successful career progression were qualified with references to discrimination within the Irish health system. Related themes of limited career progression and unclear career pathways were also frequently mentioned:

“Ireland has provided me with very good training for which I am ever grateful, but along my career I have felt discrimination and inequality.” (Survey respondent 291, 1995).

The percentage of *career-oriented migrants* who reported that their experiences of working as a doctor in Ireland had not lived up to expectations at 67 % (141/211) was slightly higher than in the overall survey population at 60 % (218/366).

### Livelihood migrant

*Livelihood migrants* cited “higher salary” as a very important factor in their initial decision to migrate to Ireland. Using this definition, *livelihood migrants* accounted for 13 % (49/366) of survey respondents. A similar proportion of qualitative respondents (3/37) were categorized as *livelihood migrants*. The *livelihood migrant* represented a smaller proportion of respondents than might have been anticipated. The *livelihood migrant* was concerned with salary levels and the relative economic advantage of working in Ireland, as these respondents demonstrate:

“As a locum GP^3^, I wish this group was exempt from taxation… the salary is not worth the living costs we are forced to pay.” (Survey Respondent 308, 2001).

“I enjoy my work as a doctor but the money for my efforts is not sufficient any more… meaning it is not worth the effort to come to Ireland to make more money than in my home country.” (Survey respondent 98, 2010).

Salary reductions following the economic recession were of concern to the livelihood migrant as Irish public sector salaries have been reduced since 2009, while personal taxation levels have increased. When compared to the wider survey population, the *livelihood migrant* was more likely, on arrival, to intend to remain in Ireland for less than 1 year – 33 % (16/49) of *livelihood migrants* compared to 14 % (53/366) of all respondents. In terms of future plans, the *livelihood migrant* was less likely than the wider survey respondents to intend to remain in Ireland permanently at 22 % (10/45) in comparison to 30 % (105/345).

### Backpacker

Glinos and Buchan [16] noted that the *backpacker migrant* works to travel and sees mobility as an opportunity to experience other countries and health systems. The *backpacker migrant* was categorized as the migrant who rated the desire to live abroad as “very important” in the decision to migrate to Ireland. Only 14 % (46/341) of survey respondents and 2/37 qualitative respondents fell into this category. However, it should be noted that only those non-EU migrant doctors working in Ireland at the time of the survey were eligible for inclusion – an unknown number of *backpacker migrant* doctors might have arrived and left Ireland without being included in the survey.

Unsurprisingly, the *backpacker migrant* was intent on a short-term stay in Ireland on arrival. However, many of those surveyed had remained for longer than intended. On arrival, only 9 % (4/46) of *backpacker migrant*s intended to remain in Ireland on a permanent basis, but at the time of the survey, 24 % (11/46) intended to do so. The following quotations from *backpacker migrants* offer an insight into the longer duration of stay:

“I met my Irish partner while here. If it were not for her, I would very likely have left after a year.” (Survey Respondent 62, 2010).

“… initially it was sort of a 6 months, you know I agreed to a 6 months and . . . just a bit more and a bit more and eventually I decided I should probably just stay here permanently.” (Doctor 38).

### Commuter

The typology categorized the *commuter migrant* as a migrant who regularly and at planned intervals commuted across borders to work [16]. *Commuter migrants* could also be considered circular migrants, return migrants [21] or temporary migrants [22], as they plan from the outset to “work for a short period of time (or repeated time-limited stretches) in the destination country in order to earn additional income, supplement pension funds or savings or improve job prospects in the home country” [21]. In this survey, respondents who indicated that they worked in Ireland for less than 12 months each year were classified as *commuter migrants* (46/366). Large numbers of doctors trained in South Africa were registered in Ireland at the time of the study [23]. They were regular commuters to Ireland, who worked as general practitioners (GPs) and contributed significantly to out-of-hours GP services [24]. Thirty-nine percent (18/46) of *commuter migrant*s identified had trained in South Africa, and 83 % (38/46) of *commuter migrants* were male.

The *commuter migrant* appeared to be more satisfied than the wider survey population, with 50 % (21/42) agreeing that their expectations of the Irish health system had lived up to the reality, in comparison to 36 % (133/366) of the wider survey population. As with the *backpacker migrant*, it is interesting to note that on arrival, only 2 % (1/46) of *commuter migrants* intended to remain in Ireland on a permanent basis. Whereas, at the time of survey, 20 % (8/41) planned to remain in Ireland on a permanent basis, perhaps indicating that temporary migration may sometimes serve as a “stepping stone” towards more permanent migration.

The qualitative case study (see below) illustrates that, despite the temporary/circular nature of their migration, c*ommuter migrants* can make a significant contribution to service delivery, while achieving their own goals (in this case, financial). A challenge associated with the category of the *commuter migrant* is that no respondent cited commuting as their primary motivation for migration. Rather, as this case study demonstrates, the primary motivation aligns this respondent as a *livelihood migrant* while migration itself followed the pattern of a *commuter migrant* (case study livelihood + commuter migrant).

**Table:** 

**Case study:** ***livelihood + commuter migrant*** Doctor 37 first came to Ireland in 2007 to work as a locum GP in an after-hours GP service and has come each year since then for “2 stints a year, of 2 and a half months or so” (Doctor 37). For Doctor 37, the availability and flexibility of working for an after-hours GP service enabled circular migration – “what attracted me was just the after-hours service… You work for a certain time and then you don’t work” (Doctor 37). Doctor 37 was of retirement age and was working in Ireland as a *commuter migrant* for financial reasons. Working as a locum GP in an after-hours GP service enabled Doctor 37 to continue working as a doctor, albeit on a part-time basis, and this was a routine that Doctor 37 was eager to continue. “If you get to that age you don’t want to… work all the time anymore because it is… a stressful job usually… but also I needed to work still for financial reasons. And I also felt like I would like to work as long as possible if my health allows it” (Doctor 37).	

### Potential returner migrants

As this study focussed on the experiences of non-EU migrant doctors in Ireland, it excluded those who had already returned home to their country of origin, as envisaged within the *returner migrant* categorization [16]. The study did, however, capture those who *intended* to return home, and given the challenges faced in seeking to conduct research on *returner migrants* [21], these are an interesting group to consider. One hundred and sixty-four survey respondents (164/366) were categorized as *potential returner migrants* in that their intention on arrival to Ireland was to return home. Sixteen percent (26/164) of *potential returner migrants* had trained in South Africa. As with the *backpacker migrants*, however, the plans of *potential returner migrants* changed over time. While, by definition, no *potential returner migrants* intended, on arrival, to remain in Ireland on a permanent basis, at the time of survey, 22 % (36/164) of them intended to remain in Ireland on a permanent basis.

Some *potential returner migrants* appear to have remained in Ireland for longer than originally intended, with 34 % (55/164) having lived in Ireland for 10 years or more at the time of survey. This perhaps indicates a change of mind or that desire to return home does not always translate into an actual return home, something that has been found in other studies [25]. These findings indicate that the desire to return home, which in some sense could be considered the most clear-cut category within the typology, is not as straightforward as it might first appear.

Some of the comments from *potential returner migrants* connected neatly with the concept of the return migrant who is preparing for return, having achieved their migration objectives [21]:

“I enjoyed it a great deal, worked hard and long hours, but Ireland was good to me. I achieved my objective of overseas experience while earning a strong currency.” (Survey Respondent 332, 2009).

Other *potential returner migrants* appeared to have had less success, perhaps indicating that sometimes the “foreign experience did not match their expectations” [16]. These respondents reported that not only had Ireland failed to live up to their expectations (they had always intended to return home) but that their experience in the Irish health system may actually have hindered return – because of limited career progression and/or de-skilling. They feared that they would be unable to re-establish themselves as medical practitioners in their home country:

“It was the most disappointing experience. I hope I will still be able to work as a doctor after all of this.” (Survey Respondent 271, unknown arrival year).

“What shall you do for doctors like me spending so many years in the Irish health system, then found themselves in a cul-de-sac and nowhere to go.” (Survey Respondent 295, 1999).

These findings offer new insight into the obstacles that *potential returner migrants* may face which may impede or delay their return.

In terms of the qualitative respondents, no respondent was categorized as primarily a *potential returner migrant*. Although many respondents expressed a desire to return to their home countries, this was generally related to the achievement of certain goals, such as the acquisition of postgraduate training, etc.

### Undocumented migrant

*Undocumented migrants* migrate for work but find themselves working below their skill level in the destination country, perhaps because of an irregular migration status and/or because their professional qualifications are not recognized [16]. When asked about their initial decision to come and work in Ireland, no respondent anticipated becoming an *undocumented migrant* in Ireland. Rather, as the case study below illustrates, migrant health workers may “end up” [16] as an *undocumented migrant* as an unintended consequence of their migration. This change in status can take its toll on the individual migrant health worker, even if it is just a step *en route* to regularization. Respondent 16 explains their experience of being undocumented as they sought to become a recognized and registered doctor in Ireland:

“By the time I had finished my exams, at that stage I had been beaten you know… I just wasn’t comfortable with my life. My ego and all that had been badly beaten. By the time I had got my first job I was not sure of anything anymore.” (Respondent 16).

Although there may be undocumented doctors in Ireland who are not practising medicine [26] or not in salaried practice, either because of an irregular migration status and/or because of difficulties having their professional qualifications recognized, for the most part, these doctors were not included in our study because they were not practising doctors.

The case study below is of one qualitative respondent working in the informal care sector while seeking to meet Irish professional registration requirements. As outlined above, becoming an *undocumented migrant* was not the primary motivation for migration for this respondent. The respondent was motivated to migrate for reasons that align with the *livelihood migrant* category and yet, on arrival, became an *undocumented migrant* while progressing through the registration process (the livelihood migrant).

**Table:** 

***The livelihood migrant (became undocumented)*** Doctor 35 is a doctor whose spouse had obtained employment in Ireland and who had reunited with his/her spouse in Ireland in 2009. At the time of interview in 2012, Doctor 35 was working as a care assistant and attending English language classes to achieve the IELTS (English language exam) with a minimum score of 7, necessary to begin the process of registering as a doctor in Ireland – “I tried to get my English better and better but… I miss my profession.” (Doctor 35). Although other doctors in similar positions had volunteered as unpaid hospital-based “clinical observers”, Doctor 35 had found this route closed – “if you want to get this volunteering job you should be insured and hospital, any hospital doesn’t want to pay extra money for only volunteering.” (Doctor 35). Doctor 35 describes the challenges for doctors seeking to join the Register of Medical Practitioners in Ireland, while meeting their own financial obligations in Ireland and sending remittances to family members back home. Doctor 35 recommends additional support for doctors in similar situations to facilitate the registration process: “If Medical Council give us opportunity or some courses especially designed for us . . . that we can get medical language… medical observation we will improve our English. We . . . are intelligent people we can and we want to do something for Ireland.” (Doctor 35).	

### Additional categories

When re-analysing the data (qualitative and quantitative) and applying it to the proposed typology [16], additional factors emerged which did not fit into any of the existing categories. These have been collated into two additional categories: the *family migrant* and the *safety and security migrant*.

### Family migrant

This category emerged initially from the qualitative data, with 9/37 respondents citing family reasons for their migration to Ireland. *Family migrants* cited family reasons as very important in their decision to migrate and accounted for 17 % (62/366) of survey respondents. Fifteen of these respondents had entered Ireland on a spouse visa (30 respondents in total entered Ireland on a spouse visa). Respondents spoke about coming to Ireland because their spouse was working or had been offered work in Ireland, while others mentioned a desire to live close to family or friends in Ireland or the UK. Fifty-two percent (32/62) of *family migrants* were female.

Sometimes, this took the form of a chain migration, that is, the formal and/or informal process through which migrants facilitate the subsequent migration of family and friends [21]. Several qualitative respondents (Doctors 8, 9, 32) mentioned the presence of family members or partners in Ireland as having had a positive impact on their integration. The process of categorizing the qualitative respondents revealed the importance of family and friendship networks in the migration decision. The decision to migrate to a specific country was frequently informed by the proximity of family and/or friends. These contacts sometimes also provided professional support in the destination country, facilitating access to employment opportunities and easing the transition to a new country.

The *family migrant* also referred to their current and future family circumstances as having an impact on their decision to stay in Ireland or migrate onwards. Sometimes, respondents were staying in Ireland because of family commitments:

“Generally I am very disappointed and would consider leaving Ireland if my husband wasn’t working here.” (Survey Respondent 358.1997).

Although family factors are important motivations for respondents, family does not appear to have a straightforward effect on the migration trajectory of respondents, with family circumstances contributing to push/pull and stick/stay factors. Family factors also come into play for respondents whose primary motivation for migration might be career, as this “Career-Oriented Migrant” explains:

“At this stage, my wife does not want to move.” (Respondent 19).

### Safety and security migrant

Another factor mentioned by two (2/37) interview respondents as an issue was the desire for improved safety and security. Thirty percent (17/57) of *safety and security migrants* had trained in South Africa. *Safety and security migrants* came to Ireland because of unrest or insecurity in their country of origin, as this respondent explains:

“I came to Ireland as a refugee. I didn’t come to Ireland as a decision to come and work. I came as a refugee, because I had a lot of difficulty back home.” (Doctor 11).

Six survey respondents reported that their immigration status on arrival was as a refugee or asylum seeker, and others who had not used formal asylum migration routes, nonetheless, regarded their migration to Ireland as forced, with one respondent describing his/her arrival status as that of “escapee” (Doctor 8). A much broader group of survey respondents (73/366) claimed that safety and security were very important motivators in their decision to migrate to Ireland. Indicating perhaps that safety and security are relative concepts. Although some migrant doctors fled persecution, a greater number opted to live and remain in Ireland because they considered it a safer and more secure alternative to their home countries. For these respondents, safety and security concerns were not the primary reason for migration, although they did contribute to the decision, as these (*career-oriented and backpacker*) respondents indicate:

“Unfortunately, the political situation back home is getting worse day by day.” (Doctor 7).

“It is a civilised environment. It is not as civilised back home.” (Doctor 12).

## Discussion

The paper set out to apply the Glinos and Buchan typology [16] to non-EU migrant doctors in the Irish context and to consider how the typology correlates with and helps to explain the experiences of migrant health workers within a destination country health system. Data from the Doctor Migration Project has validated the typology proposed by Glinos and Buchan [16]. The typology, as applied, can help destination countries to attract migrant health workers who are a better “fit” with the destination country health system and can help to inform policy and aid health human resource planners in retaining an engaged and committed [1] migrant health workforce. Finally, although an internationally relevant framework, the typology [16] can be modified to local conditions to provide valuable insights into health professional mobility from either a destination or a source country perspective.

### Migrant–destination country fit

Considering the proportion of non-EU migrant doctor respondents who were *career-oriented migrants*, the question arises as to why they were recruited to work in a health system which was seeking to fill vacancies that offered limited or no career progression opportunities [6]. In tandem with the typology of health worker mobility, perhaps a typology of destination country health systems should be developed. In such a typology, the Irish health system in this instance might be categorized as *service-oriented* or *vacancy-driven*, a system in which *career-oriented migrants* would be unlikely to prosper, but which might offer a good fit to *livelihood*, *backpacker or commuter migrants*. In this regard, perhaps the combined typologies could facilitate a better fit between migrant motivation and destination country conditions and provide a greater likelihood of “win-win” rather than “brain waste” [9] outcomes.

An example of an initiative which has sought to more closely align the expectations of migrant doctor and destination country health systems is Ireland’s International Medical Graduate Training Initiative which has benefited approximately 150 migrant doctors in Ireland since 2013 [27, 28]. The initiative recruits directly from the source country, offering doctors the opportunity to obtain postgraduate training in Ireland and then return to the source country with newly acquired skills [27, 28]. Through this programme, Ireland could be described as a *specialist training* destination country offering training to *career-oriented* and/or *returner migrants*. The typology [16] could assist health human resource managers to develop recruitment and retention policies that synchronize with the needs of the migrant health worker and those of the destination country health system.

### Retaining an engaged and committed migrant health workforce

The application of the typology provides scope for a better match between the destination country and newly recruited migrant doctors, but how might the typology be used to retain those already in post in the destination country? Categorizing the migrant doctor workforce according to their motivation should help in the development of policies to retain and motivate them. The predominance of *career-oriented migrants* among migrant doctor respondents combined with the significant overlap identified between *career-oriented* and other categories (Tables 2, 4 and 5) indicate that improved access to postgraduate training opportunities for non-EU migrant doctors already in post would be likely met with a positive response in the Irish context. The provision of postgraduate training to migrant doctors in Ireland would be a retention measure specifically targeted at this group. No specific retention measures to date have targeted migrant health workers [29]. Given that one in three doctors in the Irish health system [18] is internationally trained, perhaps a more strategic approach to their retention is justified. Although there is a need for further research on the costs of high turnover and benefits of workforce stability, “intuition would suggest that improving retention and stability of the health workforce brings benefits to staff, the organisation and those being cared for” [30]. Destination countries cannot be complacent about the stability of their migrant health workforce or about the potential costs associated with high-turnover rates.

**Table 5:** Typology and overlap (Doctor Migration Project quantitative data)

		Livelihood migrant	Career-oriented migrant	Backpacker migrant	Potential returner migrant	Commuter	Family migrant	Safety and security migrant
	Total	49	220	46	164	46	62	73
Livelihood migrant	49	–	26	14	21	12	12	21
Career-oriented migrant	220	26	–	28	89	17	29	48
Backpacker migrant	46	14	28	–	16	7	8	24
Potential returner migrant	164	21	89	16	–	28	22	20
Commuter migrant	46	12	17	7	28	–	4	11
Family migrant	62	12	29	8	22	4	–	17
Safety and security migrant	73	21	48	24	20	11	17	–

### Typology informing policy interventions

The process of applying the typology to the findings of the Doctor Migration Project also revealed the extent to which health worker motivation changes over time. The transition among some migrant health workers from short-term categories, such as *backpacker* or *potential returner migrants*, towards a desire for greater permanency in the destination country indicates the extent to which motivations can change radically over time. This signifies an opportunity for the destination country to use typology-informed policy interventions to encourage the retention of migrant health workers in the destination country.

However, what is an opportunity for the destination country can represent a challenge for the source country. The changing motivation of migrant health workers means that source countries cannot presume that all emigrant health workers will return to their country of training, even if that was their original intention, a finding reported in the literature [25] and in a forthcoming paper from this study. A better understanding of emigrant health professionals, facilitated by research and the application of the typology, would enable source countries to develop targeted policy measures to promote return.

### Modifying the typology

#### Additional categories

Although a typology cannot be comprehensive [16], it may benefit from adaptations informed by research. In relation to the data on non-EU migrant doctors in Ireland, the authors recommend two additional categories. One reflects the influence of family and friends on the migration decision (*family migrants*); the other stems from the safety and security considerations that migrant health workers factor into their migration decisions (*safety and security migrants*). As Omaswa has noted in an African context, the “most powerful incentives to international migration are insecurity, injustice, poor working conditions and economic and social strife” [31]. The *safety and security migrant* category reflects the role played by insecurity as a driver of health worker migration.

#### Ranking categories

The authors suggest another potential modification to the typology as a result of the process of applying the typology to the qualitative data. There is the potential for the typology to be “ranked”, with some categories considered primary and others considered secondary (see Table 6).

**Table 6:** Ranking the typology

Primary	Secondary
Career-oriented migrant	Commuter migrant
Livelihood migrant	Undocumented migrant
Backpacker migrant	Returner migrant
Family migrant	
Safety and security migrant	

When considering the main reason for migration, no qualitative respondent could be categorized a *commuter*, *undocumented* or *returner migrant* despite the fact that some respondents were commuting, others were undocumented and others intended to return home. However, these factors were secondary aims, co-existing alongside a desire for career progression or for postgraduate training or to achieve safety and security. In a sense, the primary aims set the respondent on a migration trajectory, while the secondary factors related to the logistics of migration (see Table 6).

Previous research by the authors demonstrated that migration decisions are not made in a vacuum but are intrinsically connected to other aspects of the health professional’s life, both personal and professional [4]. Of particular interest from a human resource management perspective is the extent to which these factors are endogenous or exogenous to the health system [4–6], as this will dictate whether retention policies should be health system focussed or whether a broader response is required. These new categories illustrate the complexity of factors influencing health workers in deciding whether to migrate onwards, remain in the destination country or return home [4].

## Conclusion

In conclusion, employing a typology of health worker migration can facilitate a more comprehensive understanding of the migrant medical workforce, a necessary prerequisite for the development of useful policy tools [16] to retain an engaged and committed [1] migrant health workforce. The findings indicate that there is some fluidity between categories, as health worker motivations change over time and also that there is overlap between categories (Table 5). This indicates the potential for policy levers to influence migrant health worker decision-making, if they are sufficiently “tuned in” to migrant health worker motivation.

### Limitations

This paper is based on non-EU migrant doctors working in Ireland. A limitation of the study is the fact that the quantitative categories are not mutually exclusive. The extent to which there is overlap between the various categories is outlined in Table 5. Although there are likely to be *undocumented migrants* in Ireland who do not practise as doctors and perhaps work in different professions in Ireland, for example, those who come to Ireland seeking asylum [26], they were not the main focus of the study. Another limitation of the project is that it is a “snapshot” of non-EU migrant doctors in Ireland at the point of interview and/or survey. As a cross-sectional study, respondents have relied on memory to recall their motives for migration and their intentions on arrival. A final limitation of the study is that it was not designed specifically around the typology [16], which was applied *post hoc*. These limitations point to areas for future research, which could include undertaking a cohort study of migrant doctors designed around the typology.

## Endnotes

^1^Including all migrant doctors (EU and non-EU) but excluding Irish-trained doctors who hold other EU or non-EU nationalities.

^2^Non-consultant hospital doctor is a junior hospital doctor in the Irish context.

^3^General practitioner

## Abbreviations

EU: European Union
GP: General practitioner
IELTS: International English Testing System
IMG: International medical graduate
NCHD: Non-consultant hospital doctor a junior hospital doctor in the Irish health system
